# Evaluation of Selected Quality Characteristics of Thin-Walled Models Manufactured Using Powder Bed Fusion Technology

**DOI:** 10.3390/ma18051134

**Published:** 2025-03-03

**Authors:** Tomasz Kozior, Jerzy Bochnia, Alicja Jurago, Piotr Jędrzejewski, Michał Adamczyk

**Affiliations:** 1 Faculty of Mechatronics and Mechanical Engineering, Kielce University of Technology, 25-314 Kielce, Poland; jbochnia@tu.kielce.pl; 2 Hydropress Wojciech Górzny, AMTH Research and Development Center, 84-230 Rumia, Poland; jurago.a@hydropress.pl (A.J.); jedrzejewski.p@hydropress.pl (P.J.); adamczyk.m@hydropres.pl (M.A.)

**Keywords:** thin-walled elements, direct metal laser sintering (DMLS), mechanical properties, surface texture, aluminum powder

## Abstract

This publication presents the results of research on selected quality features of sample models made using 3D printing technology from the Powder Bed Fusion (PBF) group and a material based on aluminum powder. Two quality areas were analyzed: tensile strength and geometric surface structure. Strength tests of thin-walled models were carried out for samples with four given thicknesses of 1, 1.4, 1.8, and 2 mm and four printing directions, namely, three in the XZ plane and one in the XY plane. The measurement of the geometric structure was carried out using optical measuring devices and by taking into account the assessment of roughness and waviness parameters. Using scanning electron microscopy (SEM), an analysis of the fracture of samples after rupture was carried out and the surface was assessed for technological defects created in the manufacturing process. The test results showed that for thin-walled sample models, there are certain technological limitations regarding the minimum sample thickness in the manufacturing process and that the strength of thin-walled models in relation to “solid” samples depends on both the sample thickness and the printing direction. Roughness parameters that determine functional quality characteristics such as friction and wear were determined and also showed a dependence on the printing direction.

## 1. Introduction

Three-dimensional printing technologies, known since the 1980s, are developing extremely dynamically, as evidenced by the development of new materials for 3D printing based on metals, plastics, and ceramics. However, numerous research works are being conducted based mainly on international standards such as ISO or ASTM, which assume a sample thickness that is usually above 2 mm. This means that the assessment of thin-walled models (below 2 mm) is omitted. Meanwhile, most models produced using 3D printing technologies have a shape and a geometry that fit into the framework of thin-walled models. This has a large application, for example, in the production of thin-walled composite models.

Thin-walled models manufactured using 3D printing technology are the subject of studies mainly in three areas: the assessment of mechanical properties [1,2,3], the aspect of cellular structures [4,5,6,7], and the assessment of utility models (prototypes) [8,9].

Bochnia et al. [3] used 3D metal powder printing technology to produce thin-walled models of samples from maraging steel (MS1) with a thickness of 0.35 to 0.6 mm in two orientations on the build platform. Tensile strength tests showed that thin-walled models were characterized by lower tensile strength than solid models by over 30% (value declared by the manufacturer). Moreover, differences in tensile strength of over 19% were recorded between samples of different thicknesses, which indicates high anisotropy of mechanical properties dependent on sample thickness and printing direction.

In the publication [6], the authors comprehensively examined the models of samples manufactured using the Selective Laser Melting (SLM) technology from a material based on 316 steel. The scope of the research included the assessment of both the technological parameters recommended by the 3D printer manufacturer and their modification in order to determine the properties of the manufactured thin-walled models. It was determined that increasing the energy density can significantly improve the technological quality of the surface layer by reducing the roughness parameters and porosity, which is extremely important in the aspect of manufacturing thin-walled models. The heat treatment applied after the manufacturing process was also assessed.

Three-dimensional printing technologies based on metal powders seem to have the greatest practical application in connection with the ongoing industrial transformation 4.0, especially in the machine industry. In the case of such applications, 3D printing is successfully used for the production of, for example, power transmission components [10], tools in the injection molding process (conformal channels), and power hydraulics components [11].

Components for power hydraulics are usually produced with milling machines, lathes, casting, or laser cutting. These methods are optimal for several established products, but each modification to the design can take up to 30 days to implement [11]. Hydraulic manifolds, for instance, are usually in the form of a block with milled perpendicular channels. This solution leaves out a lot of spare materials around the channels, increasing the volume and mass of such manifolds. Moreover, perpendicular channels are not optimal for fluid flow, but traditional milling does not allow for much optimization of channel geometry. For more complex manifolds, milling alone can be a very costly process, requiring many operations and multiple tools. Additive manufacturing allows for geometrical optimization, which was not possible with traditional methods. Cooper et al. [12] proved that a thin-walled (0.5 mm) hydraulic channel produced from titanium Ti64 powder can withstand a pressure as high as 25 MPa, resulting in 250% efficiency improvement. Matthiesen et al. [13] produced an optimized pump housing from 316 L, reducing its volume to 48.5% compared to the original design. Wiberg et al. [14] also conducted a study that validated the advantages of an additively manufactured high-pressure hydraulic pump produced from aluminum with a wall thickness of 3 mm, which was able to withstand pressures as high as 50 MPa and has a 45–80% reduction in pressure loss and 35% reduction in mass.

Three-dimensional printing technologies based on metal powders according to ISO/ASTM 52900 [15] are mainly classified as Powder Bed Fusion (PBF). The most commonly used materials in this group of technologies include popular tool steels as well as materials based on titanium, aluminum, copper, nickel, and tungsten. Aluminum-based materials are used where the weight-to-strength ratio is crucial and where the functional properties of aluminum are irreplaceable. These materials are often subjected to both heat treatment and coating, which, given the high roughness in 3D printing, is a reasonable procedure that affects not only the improvement in surface roughness parameters but also changes in functional features, such as surface hydrophobicity. Madej et al. [16] conducted a comprehensive study on coatings applied to materials used in 3D printing for medical applications, Ti6Al4V, where the authors analyzed the hardness and the surface and tribological properties of the produced coatings. It was determined that the use of TiCN coatings increases hardness and wear resistance, which may have large potential benefits in the case of applications such as power hydraulics. In the case of coatings, as shown by Kowalczyk et al. [17], the surface condition of the samples expressed both by roughness and porosity determines the properties of the applied coating; hence, the research in the presented work can also be used at a later stage of coating production in models produced by 3D printing using materials based on aluminum powder and that are covered with coatings.

Our previous research proved that thin-walled models manufactured using 3D printing technologies, both from materials based on metal powders and plastics, are much more sensitive to all types of surface defects as well as the technological process itself, which is designed for solid-type models with at least several dozen layers of joined material.

This publication describes the research on thin-walled models produced using 3D printing technology for applications in power hydraulics. The technological limitations of producing thin-walled structures and the influence of the printing direction on the mechanical properties and quality of the technological surface layer were analyzed. The research results can be used in the design of a technological process for producing hydraulic blocks with reduced mass and optimized flows.

## 2. Materials and Methods

### 2.1. Materials

The material used for sample manufacturing was aluminum alloy AlSi10Mg (EOS GmbH, 82152 Krailling, Germany), with the composition as shown in Table 1. The powder was spherical in shape with a generic particle size distribution of 25–70 µm.

According to the datasheet provided by EOS, samples in the as-manufactured state had an average defect percentage of 0.2% and average density of 2.65 g/cm^3^. Other typical mechanical properties are listed in Table 2. These results are specifically for the machine and layer thickness of the samples in the as-manufactured state that were used in this study.

### 2.2. Methods

**Figure 1:**
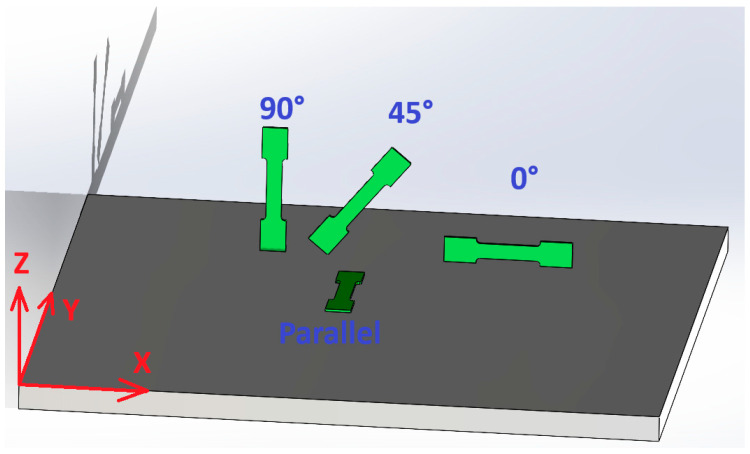
Orientation of sample models on the build platform.

**Figure 2:**
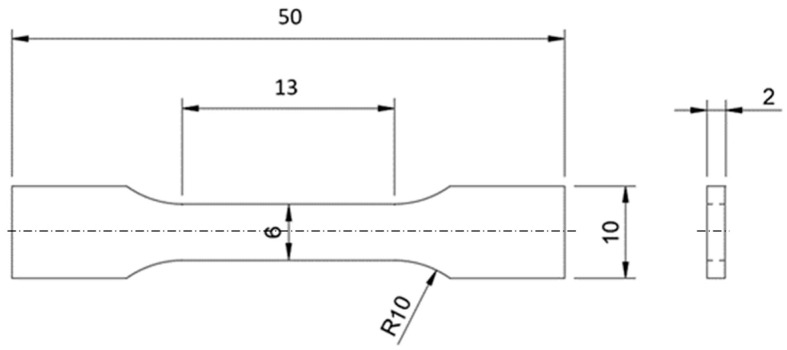
Dimensions of test samples with 2 mm thickness.

In the conducted tests, the 3D printing technology from the Powder Bed Fusion group was used to manufacture the samples. The samples were made in four variants of the location on the building platform: three in the XZ plane and one in the XY plane, as shown in Figure 1. The shape of the samples is shown in Figure 2, where the dimension of 2 mm (thickness) was changed, depending on the type of sample, to 1 mm, 1.4 mm, 1.8 mm, and 2 mm. The samples were marked by the code D-T-K, where D is the printing angle (0°, 45°, 90°, or FD (parallel to the building platform)), T is the thickness of the sample (1, 1.4, 1.8, and 2.0 mm—labeled as 1, 14, 18, and 2, respectively), and K is the sample number in the series from 1 to 5. For example, samples with a thickness of 2 mm and a printing angle of 90° were described by the code D90-T2-1. The parallel sample was marked by the letter F, e.g., FD0-T2-1. The samples were shaped according to EN 10002-1:2001 (Metallic materials—Tensile testing—Part 1: Method of test at ambient temperature).

#### 2.2.1. PBF Technology

The machine used for sample manufacturing was EOS M400 (EOS GmbH, Germany), equipped with a 1-kW ytterbium laser. All process parameters were left unchanged from the standard parameters set by the manufacturer to simulate normal work conditions. One exception was the differential pressure setting because the standard setting of 3.5 mbar tends to blow the light aluminum powder away from the platform, resulting in lower density builds near the inert gas inlet. To avoid these defects, the pressure difference was reduced to 2.8 mbar (the minimum value allowed by the manufacturer). All other machine settings are listed in Table 3.

**Figure 3:**
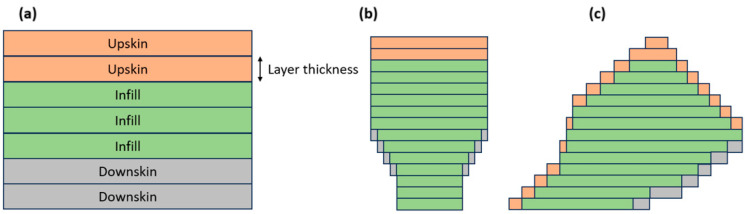
Three printing zones (upskin, infill, and downskin) represented on (**a**) a parallel sample, (**b**) a vertical sample, and (**c**) a sample at a 45° angle.

In this study, it is important to distinguish three exposure zones: upskin, infill, and downskin (see Figure 3) because in thin-walled structures, they have a significant impact on the overall structural integrity. Upskin and downskin were set to two layers, and both had double exposure, meaning the laser was scanning the top and bottom surfaces twice because it usually achieves better quality (default setting).

#### 2.2.2. Tensile Test

The static tensile test was performed using a Shimadzu MWG-SFL-20 kNA (Shimadzu Corp., Japan) testing machine. The tensile test was performed at a set speed of 0.2 mm/min. During the measurement, the stress–strain diagram was recorded after previously entering the dimensions “a” and “b” into the machine’s computer program, which were different for each sample.

#### 2.2.3. Surface Texture Measurement

**Figure 4:**
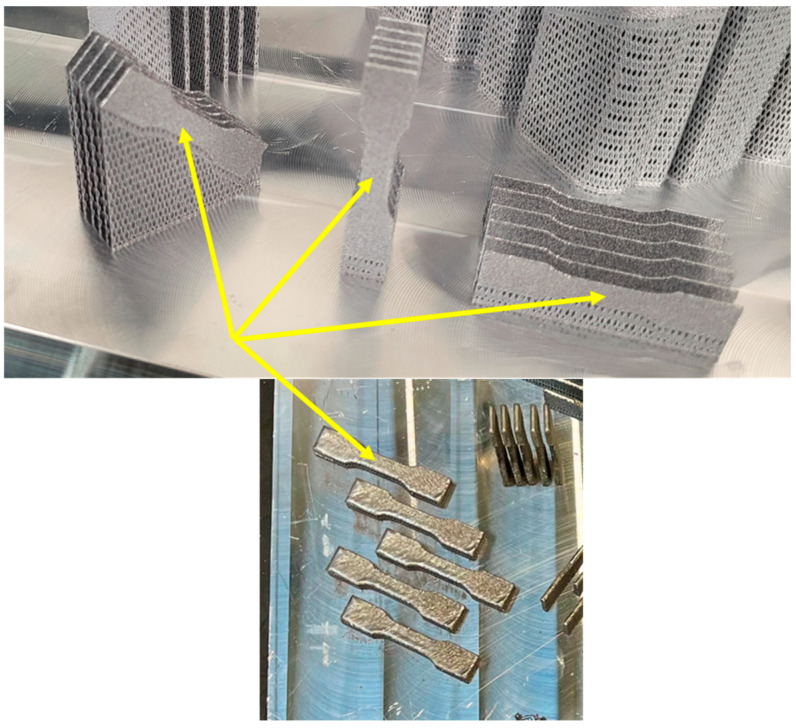
Area of measurement of the surface texture.

The measurements of the geometric structure of the surface were carried out using a Leica DCM8 (Leica Camera, Germany) microscope. The device allows for the measurement of the geometric structure of the surface using three research methods: interferometry, focus variation, and confocal microscopy. In this study, the focus variation method was used to measure all samples due to the fact that for the analyzed material, based on preliminary studies, it was found that for this material, this method is characterized by the smallest number of so-called unmeasured measurement points. The measurement was carried out on the flat surface of the samples at the locations marked in Figure 4. In this study, a lens with x5 magnification was used (measured area of 3.4 *×* 2.6 mm^2^), and the λc, λs, and λf filtration processes were carried out in order to obtain roughness and waviness profiles. The spatial roughness parameters were determined based on the ISO 25178-2 standard [18], and the profile parameters were obtained according to ISO 21920-2 [19]. During both the initial evaluation of the measurement method and further analysis of the test results, attention was paid to the so-called number of unmeasured points (NMP), which has a direct impact on the reliability of the determined surface parameters [20,21].

#### 2.2.4. Contact Angle Measurement

The contact angle measurement was performed on the surface of the samples in order to determine the functional properties of the aluminum material, which is an important functional quality parameter. The Theta Flex Optical Tensiometer (Biolin Scientific AB, Sweden) was used to perform the contact angle measurement. The measurement was performed using 5 µL of engine oil (5W40) for a single drop.

#### 2.2.5. Microstructure Analysis

Microscopic examination of the fracture surface of the samples as well as the analysis of powder morphology images before and after the sintering process were performed using the JEOL JSM-7100F scanning microscope (SEM, JEOL, Tokyo, Japan). Several levels of magnification, ranging from ×200 to ×2000, were used.

## 3. Results and Discussion

### 3.1. Dimensional Accuracy

Table 4 presents the results of the dimensional measurements of the static tensile test conducted on the manufactured samples. The thickness and width of each sample were measured in three locations in the measurement zone, and the calculated average value of the three measurements was recorded (Table 4). The measurement was made using an electronic micrometer with an accuracy of 0.01 mm. The columns contain sample identifiers (column No.) and sample dimensions (thickness—a (mm) and width—b (mm)). The nominal thickness is the value that was assigned during the design in the 3D CAD program. The thicknesses of the manufactured samples and other dimensions differ from the CAD designed values.

A very important problem that appeared during the production of samples was differences in the dimensions of thickness and width compared to those specified in the CAD models set on the virtual building platform. The largest differences in the obtained dimensions (see Table 4) concern the measurement series with a thickness of 1 mm, where, in three cases, the dimensional difference for the dimension “a” (thickness) was as much as 57% compared to the nominal CAD dimension. The dimensional differences in the thickness “a” for samples with a thickness of 0.5 mm ranged from 26% to 34%. In the remaining cases, the thickness “a” of the samples was characterized by a deviation between 10% and 24%. The most accurate samples were those made with a specified thickness of 2 mm, where the deviations were 8.75% on average (from 7% to 12%). The measurement of the width of the samples (dimension “b”) shows that the samples made with the specified orientation (FD) on the building platform are characterized by the smallest deviations compared to the nominal CAD dimension, which was below 0.79%. Slightly less accurate sample execution was recorded for the variant of the position on the construction platform at 90°, where the dimensional deviations amounted to an average of 1.47%. For the other two variants, 0° and 45°, the dimensional deviations were 7.6% and 8.77%, respectively.

### 3.2. Tensile Test Results

**Figure 5:**
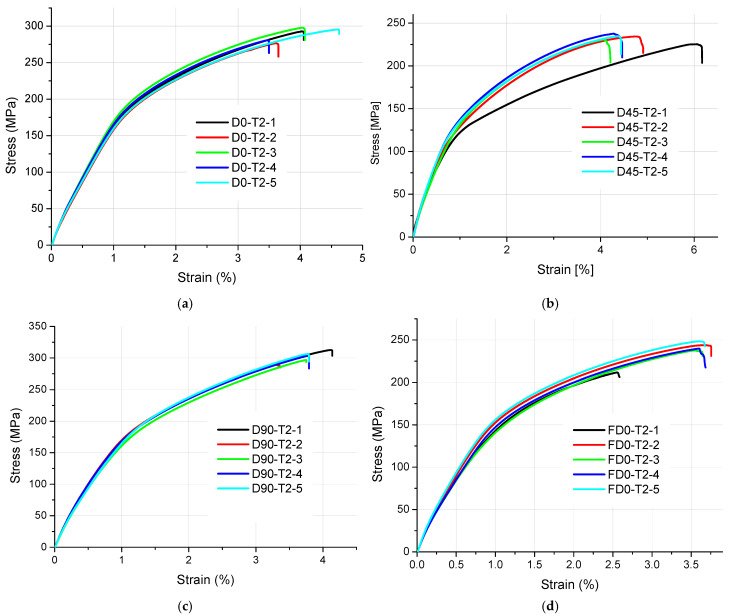
Tensile stress–strain curves of 2 mm thick samples made of AlSi10Mg aluminum alloy powder: (**a**) orientation: 0°, (**b**) orientation: 45°, (**c**) orientation: 90°, and (**d**) orientation: flat (0°).

**Figure 6:**
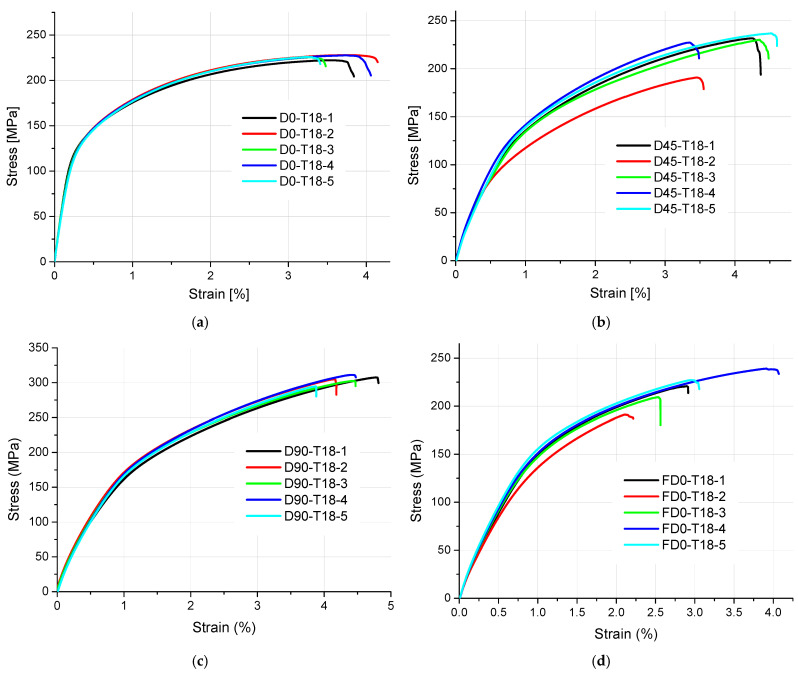
Tensile stress–strain curves of 1.8 mm thick samples made of AlSi10Mg aluminum alloy powder: (**a**) orientation: 0°, (**b**) orientation: 45°, (**c**) orientation: 90°, and (**d**) orientation: flat (0°).

**Figure 7:**
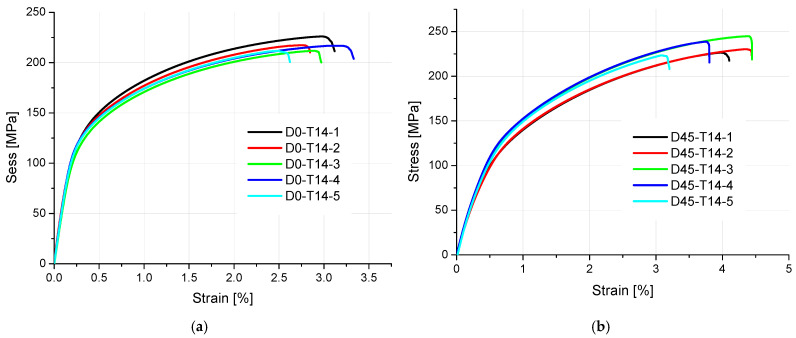

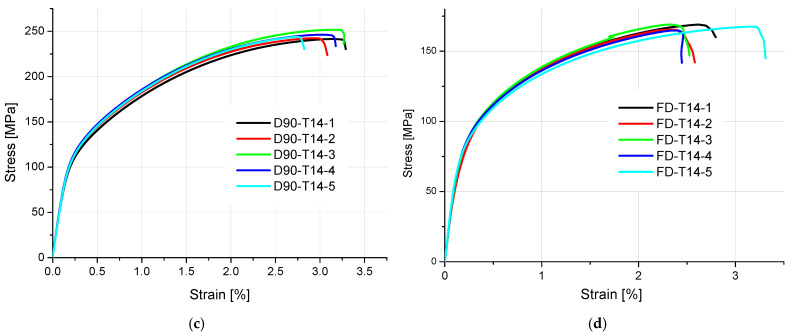
Tensile stress diagrams of 1.4 mm thick samples made of AlSi10Mg aluminum alloy powder: (**a**) orientation: 0°, (**b**) orientation: 45°, (**c**) orientation: 90°, and (**d**) orientation: flat (0°).

**Figure 8:**
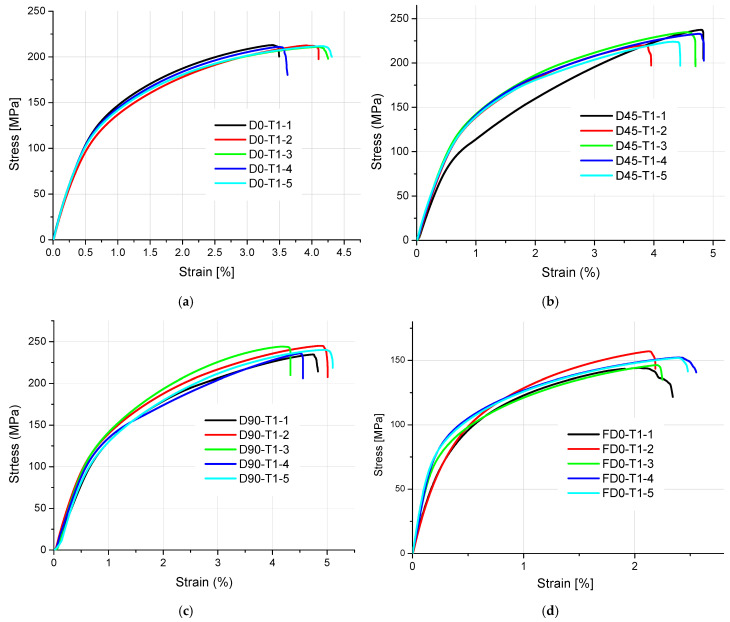
Tensile stress–strain diagrams of 1 mm thick samples made of AlSi10Mg aluminum alloy powder: (**a**) orientation: 0°, (**b**) orientation: 45°, (**c**) orientation: 90°, and (**d**) orientation: flat (0°).

**Figure 9:**
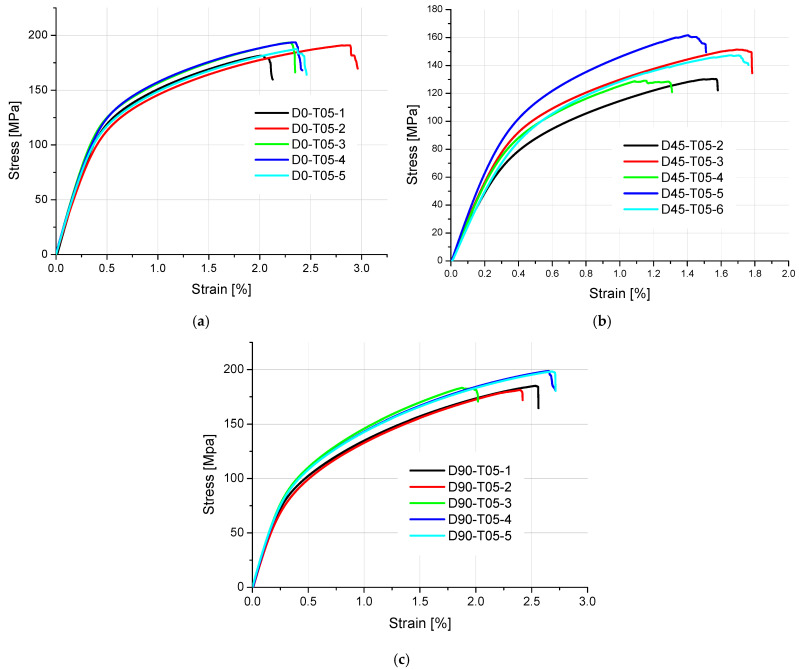
Tensile stress–strain curves of 0.5 mm thick samples made of AlSi10Mg aluminum alloy powder: (**a**) orientation: 0°, (**b**) orientation: 45°, and (**c**) orientation: 90°.

**Figure 10:**
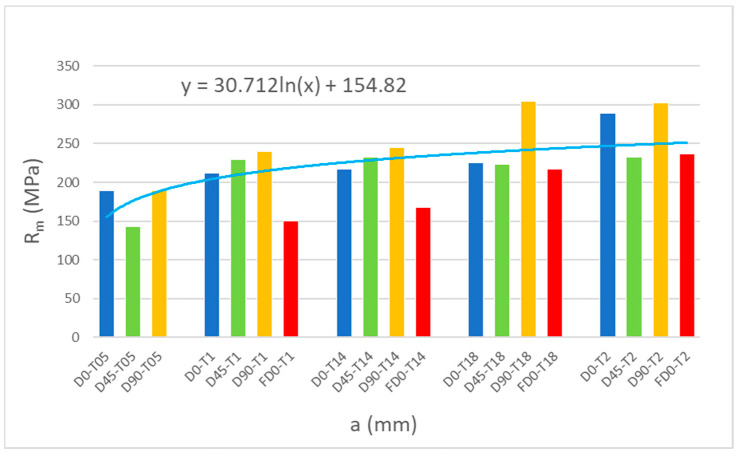
Relationship between the tensile strength of samples made of AlSi10Mg aluminum alloy powder and the thickness and orientation of the printing platform.

**Figure 11:**
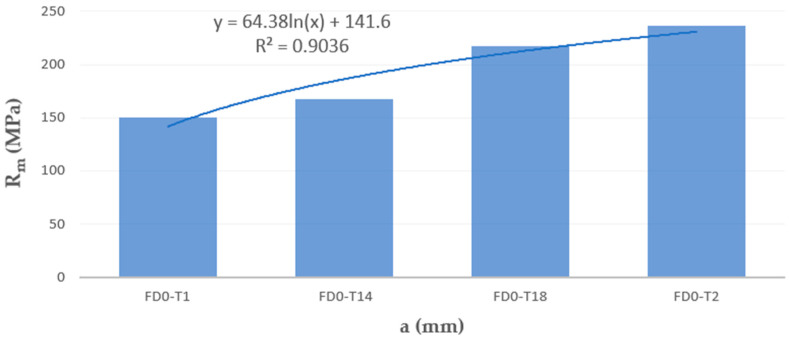
Dependence of the tensile strength of samples made of AlSi10Mg aluminum alloy powder on their thickness for the FD orientation.

The results of tensile strength and surface texture for all analyzed samples are presented below in Figure 5, Figure 6, Figure 7, Figure 8 and Figure 9 and Table 5. The relationship between the tensile strength of the samples and the thickness and orientation of the printing platform is given in Figure 10, and the dependence of their tensile strength on their thickness for the FD orientation is given in Figure 11.

The effect of sample thickness on tensile strength is shown in the bar graph in Figure 10, and a detailed relationship for parallel (flat) samples (FD) is shown in Figure 11.

Analyzing the above figures and graphs, a trend of increasing tensile strength with increasing sample thickness was observed. This increase may be logarithmic, as indicated by the added trend line. We observed a similar phenomenon in a previous study, where the tensile strength of Ti-6Al-4V alloy samples with thicknesses of 0.5, 1, 1.5, 2, 3, and 6 mm was tested [22]. In that work, it was found that increasing the sample thickness above 2 mm did not significantly affect the tensile strength. However, the results obtained for samples with thicknesses ranging from 0.5 to 2 mm indicate a significant increase in tensile strength.

Observing the tensile stress curves shown in Figure 5, Figure 6, Figure 7, Figure 8 and Figure 9, it can be seen that the tensile samples, which were manufactured in the 45° orientation, show greater dispersion of the strength. This is also confirmed by the values of the standard deviation given in Table 5, which were highest in all series of samples with nominal thicknesses of 0.5, 1, 1.4, and 1.8 mm. The largest SD was 18.47 MPa, with an average strength (Rm) of 223.4 MPa for the D45-T18 sample series. For all the remaining measurement series, the SD ranged from 0.84 MPa for the D0-T1 series to 18.01 MPa for the FD0-T18 series.

The dependence of the tensile strength of the samples on their thickness for the so-called flat orientation (FD) is presented separately in Figure 11. Among all printing directions, the series of FD samples achieved the lowest strength values while maintaining the trend of increasing strength with increasing sample thickness, which can be approximated by a logarithmic trend line.

Analyzing the results of strength tests, it can be stated that in industrial practice, when designing thin-walled elements, for example, for hydraulic applications, or when manufacturing thin-walled structures of medical implants and medical fillings, it is necessary to take into account the need to increase the safety factor and appropriate topological optimization adapted to the requirements of 3D printing. For example, samples manufactured with a given thickness of 1 mm are characterized by only 73.8% of the strength of solid-type samples, i.e., those manufactured with a given thickness of 2 mm (Table 5). In such cases, when designing structures, an increased safety factor of 1.36 should be introduced.

The least recommended printing direction for thin-walled elements based on the presented mechanical test results is the flat printing direction, i.e., samples marked with the symbol F, e.g., FD0-T2-1. For this location variant, it was not possible to make the thinnest samples with a thickness of 0.5 mm. On the other hand, FD samples with a thickness of 1 mm showed lower Rm than samples manufactured in the other location variants. The tensile strength of the FD samples was only about 62% of the tensile strength of the samples manufactured with a given angle of 90°. In the case of samples with a thickness of 1.4 mm, the tensile strength of the F samples was only 67% of the tensile strength of the samples manufactured with a given angle of 90°. For samples with a thickness above 1.4 mm, i.e., 1.8 mm and 2.0 mm, the tensile strength was at a similar level for all analyzed printing directions, as shown in Figure 11.

### 3.3. Surface Analysis

**Figure 12:**
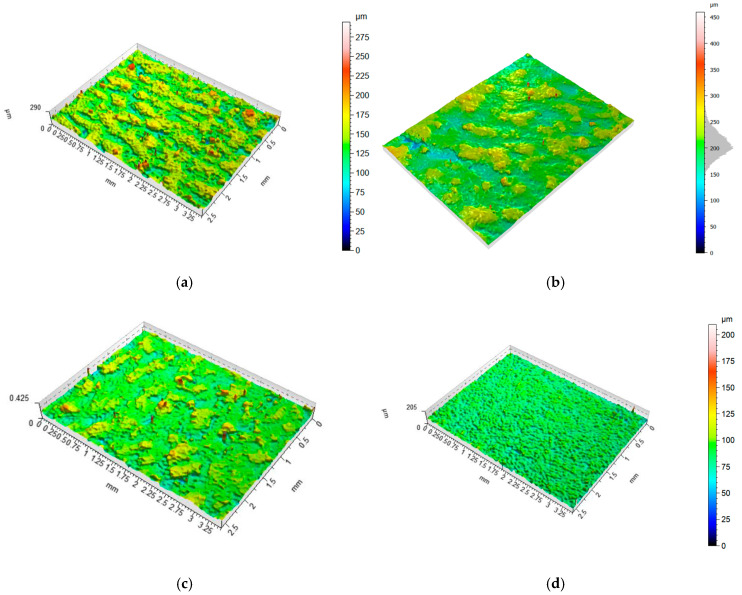
View of a 2 mm thickness sample surface produced with four given printing directions: (**a**) 0°, (**b**) 45°, (**c**) 90°, and (**d**) FD (samples parallel to the building platform).

Table 6 shows the determined surface roughness and waviness parameters. In addition, Figure 12 shows the surface view for different printing directions.

By analyzing the number of unmeasured points (NMP) for all samples, which is presented in Table 6, it can be stated that the measurement performed using the focus variation method shows extremely high reliability. In the case of all surfaces, the NMP was below 1%.

**Figure 13:**
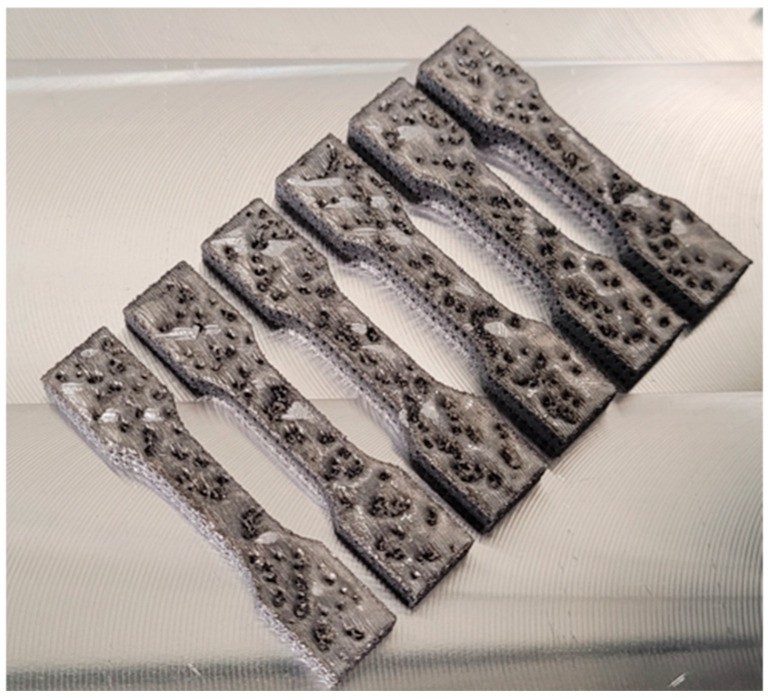
Surface view of samples of FD0-T05, which could not be fabricated.

Based on the evaluation of the 2D roughness parameter (Ra), it can be stated that the evaluation of this parameter (which is the simplest one) alone provides a lot of valuable information about the surface quality of individual samples. The calculated average value of the Ra parameter for all nineteen samples was 10.66 µm. However, a deeper analysis shows a large diversification of the results. The average roughness (Ra) value for samples manufactured with the specified angles of 0°, 45°, and 90° were 11.33 µm, 10.81 µm, and 11.65 µm, respectively. The Ra parameter for samples made parallel to the build platform for three thicknesses, i.e., 1.4 mm, 1.8 mm, and 2 mm, was characterized by an average value of Ra (PD) 4.15 µm, which is only 39% of the average value of the Ra parameter for all analyzed samples. As shown in Figure 13, it was not possible to make samples parallel to the build platform with the given thickness of 0.5 mm, and the samples with a thickness of 1 mm were characterized by a very high value of the Ra parameter of over 20 µm; hence, it can be assumed that in the presented 3D printing strategy, it is not recommended to produce thin-walled samples with a thickness below 1 mm if the goal is to obtain high surface quality. Based on the analysis of the spatial roughness parameter (Sa), it can be seen that its value is characterized by the same tendency as for the profile parameter Ra. In the case of waviness parameter (Wa), it can be seen that higher values are obtained for thin-walled samples. The average value for samples in the range of 1–1.4 mm and the three printing angles (0°, 45°, and 90°) is 13.62 µm, and for samples above this thickness, the average value is 11.77 µm, which is 14% lower.

Further evaluation of the surface quality by analyzing the Rsk parameter also provides valuable information about the symmetry of the profile ordinate distribution relative to the mean line. The value of this parameter is positive in most cases (16 samples), and negative only in three cases. It can be concluded that the surface is mostly characterized by peaks with a sharpened geometry and that the depressions have wide gaps. Such an evaluation provides a lot of information about the functional quality features of the products, among others, related to the material’s resistance to wear and the ability of the surface to transfer contact loads and conductivity.

### 3.4. Contact Angle Measurement

**Figure 14:**
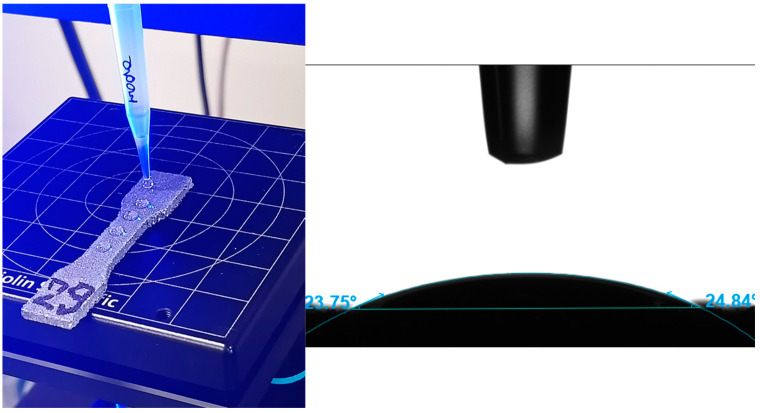
Contact angle measurement—left view, with an example graph for both angles—right view.

**Figure 15:**
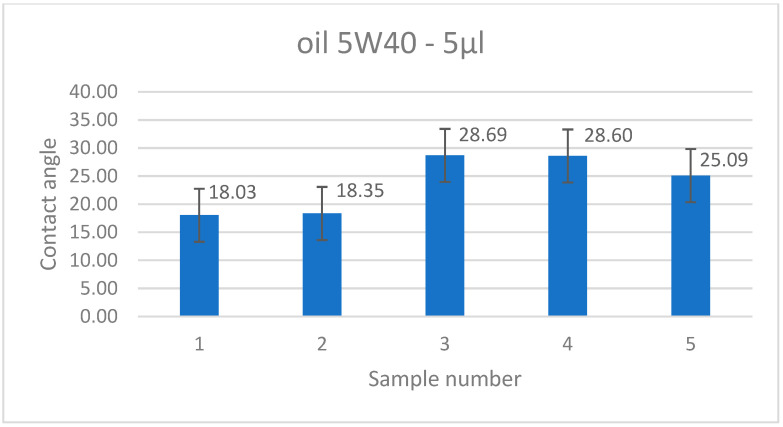
Contact angle measurement result for all measurements.

Figure 14 shows an example of the contact angle measurement, and Figure 15 shows the average values of the determined angle for five measurements of 2 mm thick samples, with the models positioned parallel to the 3D printer’s working platform during production.

The contact angle measurements ranged from 18.03° to 28.69°. Both the shape of the drop shown in Figure 14 and the measured values indicate the hydrophilic properties of the tested liquid. The contact angle values may be important in assessing the flow of oil through channels of various hydraulic devices, e.g., valves, distributors, etc. Bailey et al. [23] presented an extensive and interesting study on the contact angle for AlSi10Mg alloy obtained by Laser Powder Bed Fusion (LPBF) and water for different orientations on the build platform of printed samples. The study showed that the contact angle was anisotropic and was observed partly due to the non-uniformity of the surface created in the LPBF process. The powder-derived AlSi10Mg alloy, typically hydrophobic, showed predominantly hydrophilic behavior at build platform orientation angles of 0° and 60°, a mixture of hydrophobic and hydrophilic behavior at orientation angles of 10° and 20°, and hydrophobic behavior at orientation angles of 30°, 40°, and 50°.

### 3.5. Microscopy Analysis

#### 3.5.1. Powder

**Figure 16:**
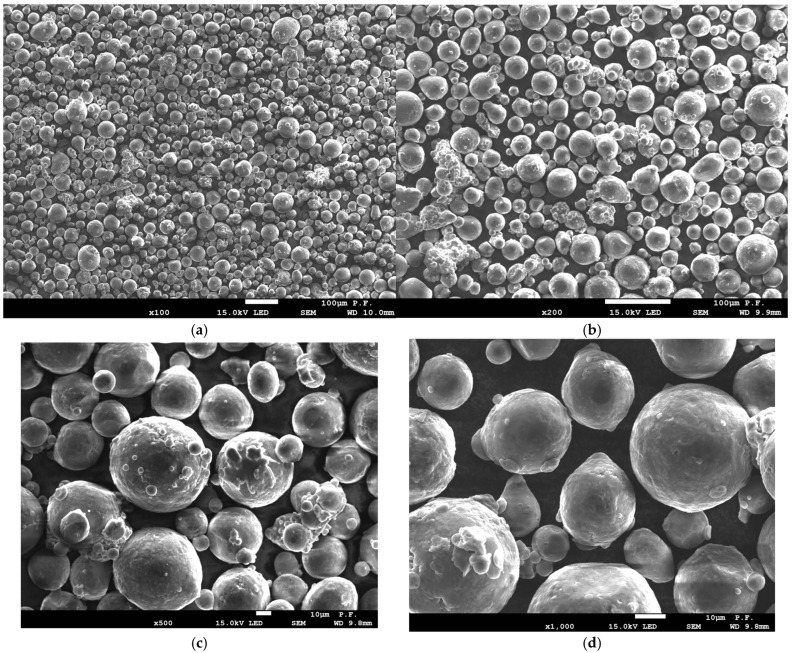

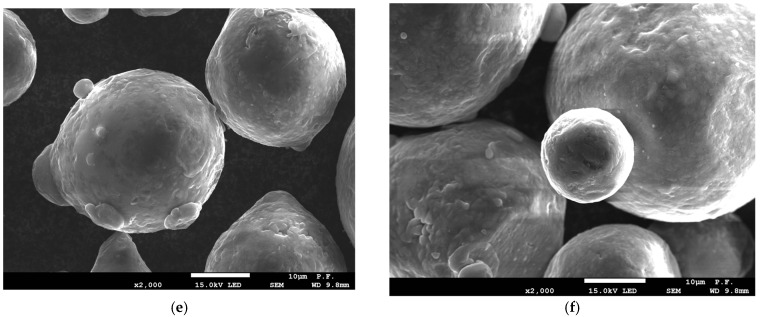
Morphology of powder used for sample production before the melting process: (**a**) ×100 magnification, (**b**) ×200 magnification, (**c**) ×500 magnification, (**d**) ×1000 magnification, and (**e**,**f**) ×2000 magnification.

**Figure 17:**
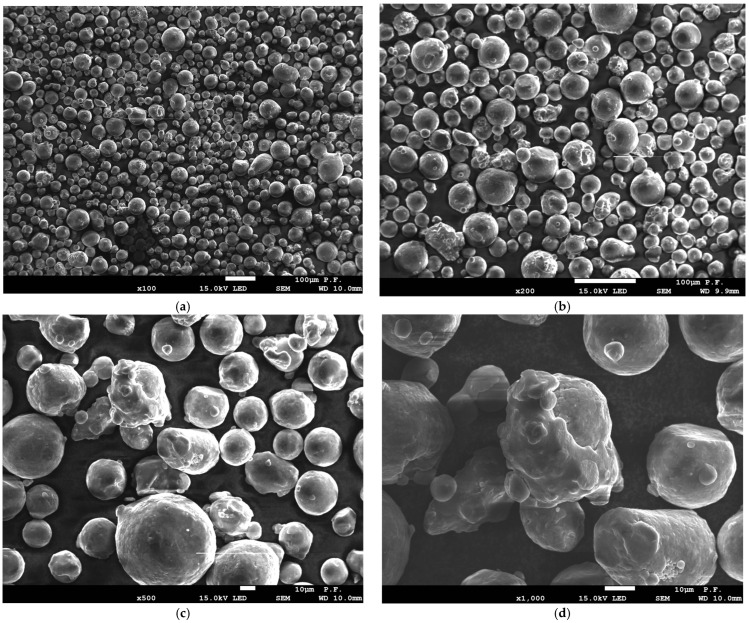

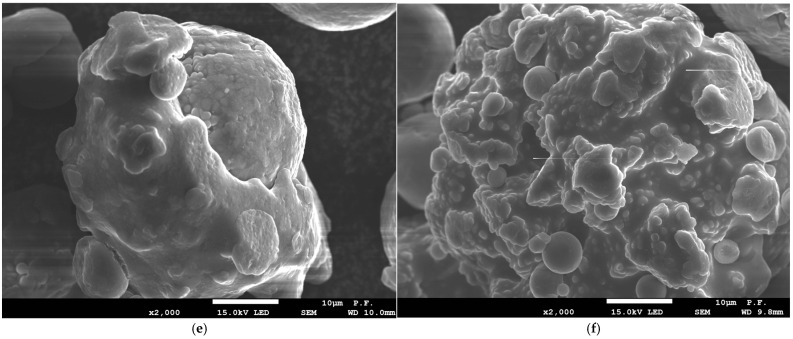
Morphology of the powder used to produce the samples after the melting process: (**a**) ×100 magnification, (**b**) ×200 magnification, (**c**) ×500 magnification, (**d**) ×1000 magnification, and (**e**,**f**) ×2000 magnification.

From the microscopic images of powder before and after the sintering process, presented in Figure 16 and Figure 17, clear differences in the powder morphology can be seen. The powder before remelting has undisturbed spherical shapes and does not show coagulation. The purity of individual powder particles is best shown in Figure 16e,f. The situation is different in the case of the powder remaining after the remelting process, which was used in further production cycles. The powder particles show a tendency to coagulate, as shown in Figure 17d,f. On the surface of the powder, we can see other smaller powder particles that are “glued” and by-products that are formed in the remelting zone. We can also see deformed powder particles that no longer have a spherical shape (Figure 17d). This type of powder particle with disturbed geometry will participate in further laser remelting until it significantly deteriorates the quality of the models produced.

#### 3.5.2. Sample Microstructure Analysis

**Figure 18:**
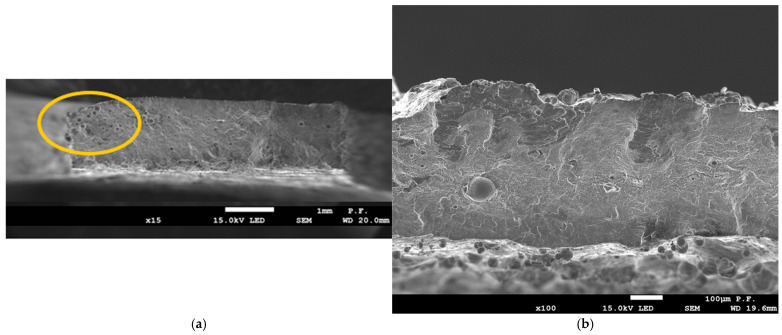

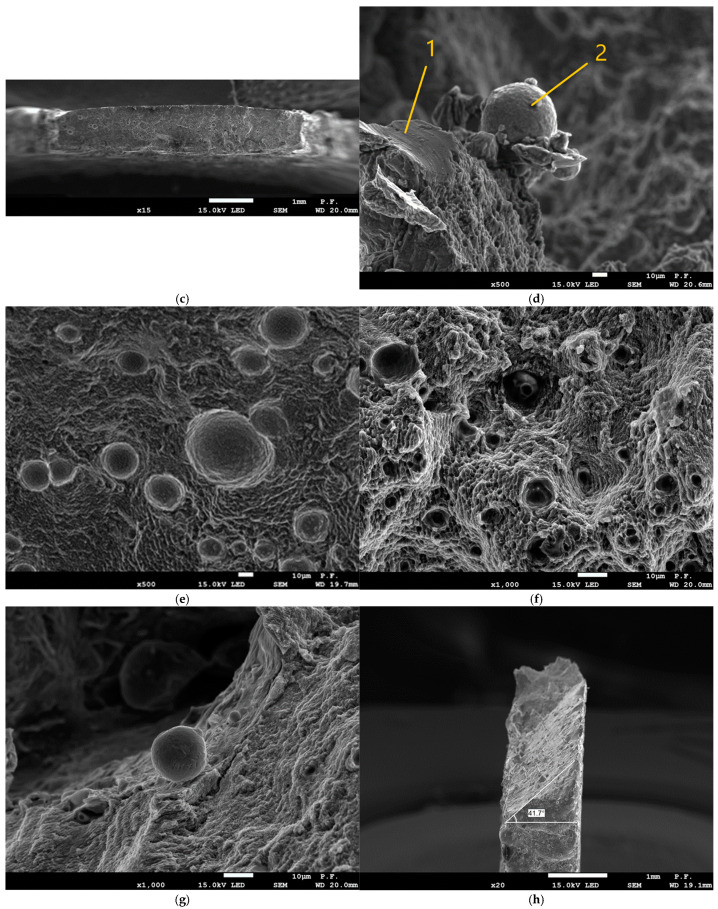

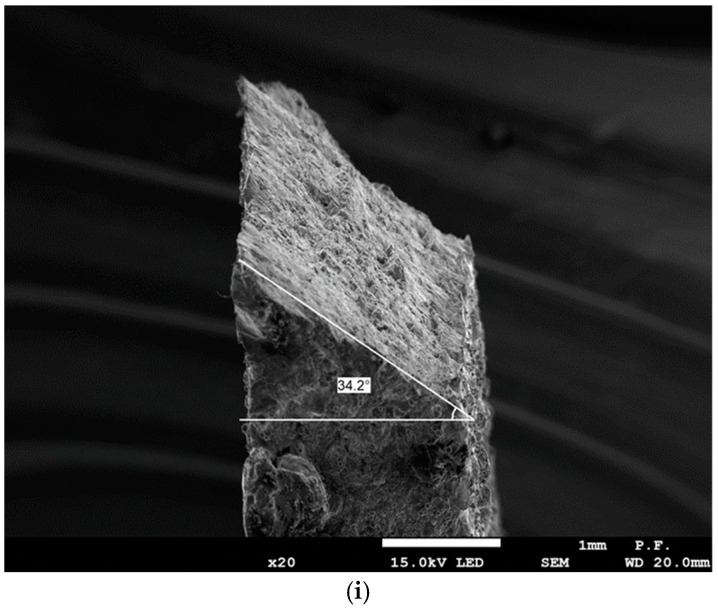
Microscopic photographs of sample fractures: (**a**) sample FD0-T14 series: ×15 magnification, (**b**) sample D0-T05 series: ×100 magnification, (**c**) sample FD0-T1 series: ×15 magnification, (**d**) sample FD0-T1: ×500 magnification, (**e**) sample FD0-T1: ×500 magnification, (**f**) sample D45-T2: ×1000 magnification, (**g**) sample D0-T05: ×1000 magnification, (**h**) sample D90-T14: ×20 magnification, and (**i**) sample D0-T05: ×20 magnification.

Figure 18 shows microscopic photos of tensional fractures of the samples. The fracture character, fracture angles, and various quality defects are visible.

All the broken samples showed a fracture at an angle of thirty to forty-five degrees, as shown in Figure 18h,i. In the upper part of Figure 18a (marked in yellow), typical defects created during the manufacturing process can be seen, such as gassing or voids resulting from unmelted powder particles. Similar phenomena also occur in metal casting processes. Laser melting in the area of the small “lake” that is being formed can be classified as micro-metallurgy. Sample D0-T05 (Figure 18b) shows a material defect that constitutes 5–10% of the “a” dimension of the sample. Such defects cause stress concentration during stretching and can reduce the strength of the sample, especially that of thin-walled elements. Figure 18c shows a fracture with roundings on the edges created after exceeding the tensile strength limit. Laser-melted materials do not behave like typical ductile polycrystalline materials, and a classic neck is not formed during stretching. In Figure 18d, for sample FD0-T1, a residue of unmelted coagulated powder can be seen at location 1 and unmelted powder after gasification can be seen at location 2, which reduces the strength properties of thin-walled samples due to the lack of melting. Figure 18e,f shows the residue of unmelted powder filling the cavities and craters, which affect the reduction in mechanical properties. Figure 18g also shows a surface with visible unmelted powder particles supported from below by pouring smaller powder particles melted in the lower part, thus reducing the strength.

## 4. Conclusions

The conducted research on the mechanical properties and surface texture for different orientations on the building platform (printing angle) of thin-walled samples allowed for the formulation of the following conclusions:

1. For thin-walled sample models, there are certain technological limitations regarding the minimum thickness in the manufacturing process, e.g., FD0-T1, for the 0° direction of flat samples (FD0) and a wall thickness of 0.5 mm; thus, it was not possible to produce these samples.
2. The strength of thin-walled models in relation to solid-type samples depends on both the sample thickness and the printing direction, which is graphically shown in Figure 10.
3. The tensile samples, which were manufactured in the 45° orientation, show greater dispersion of strength, which was also confirmed by the values of the standard deviation shown in Table 5, which reached the highest values in all series of samples with nominal thicknesses of 0.5, 1, 1.4, and 1.8 mm. The largest SD was 18.47 MPa, with an average strength (Rm) of 223.4 MPa for the D45-T18 sample series.
4. During the measurements of the sample geometry before the static tensile test, differences in the thickness and width dimensions were found in relation to those designed in CAD models and set on the virtual construction platform. The largest differences in the obtained dimensions concern the measurement series with a thickness of 1 mm, where, in three cases, the dimensional difference for the “a” dimension, i.e., thickness, was as much as 57% in relation to the nominal CAD dimension. The dimensional differences in the “a” thickness for samples with a thickness of 0.5 mm ranged from 26% to 34%. In the remaining cases, the “a” thickness of the samples was characterized by a deviation between 10% and 24%. The most accurate samples were those made with a given thickness of 2 mm, where the deviations averaged 8.75% (from 7% to 12%).
5. Anisotropy in the surface of samples was found. The average roughness values for samples made with the specified angles of 0°, 45°, and 90° were 11.33 µm, 10.81 µm, and 11.65 µm, respectively.
6. Both the shape of the drop shown in Figure 14 and the measured values of the contact angle indicate the hydrophilic properties of the tested material.
7. Based on microscopic observations, a few occurrences of material defects in the laser melting process were found, such as the residue of unmelted powder and the occurrence of gassing, craters, and inclusions, which can reduce the strength properties of thin-walled samples.

## Figures and Tables

**Table 1:** Material composition of AlSi10Mg.

Elements	Wt (%)
Al	balance
Si	9.0–11.0
Fe	≤0.55
Cu	≤0.05
Mn	≤0.45
Mg	≤0.2–0.45
Ni	≤0.05
Zn	≤0.10
Pb	≤0.05
Sn	≤0.05
Ti	≤0.15

**Table 2:** **Selected mechanical properties of** material AlSi10Mg.

	Yield Strength Rp0.2 [MPa]	Tensile Strength Rm [MPa]	Elongation at Break A [%]
**Vertical**	240	380	2
**Horizontal**	260	400	3

**Table 3:** Process parameters.

Parameter	Value
Layer thickness	90 μm
Platform temperature	165 °C
Atmosphere	Nitrogen
Differential pressure	2.8 mbar
Gas flow	300 m^3^/h
Recoating speed	250 mm/s
Laser power	600 W
Wave type	Continuous
Infill/downskin hatch distance	160 μm
Upskin hatch distance	200 μm
Infill laser speed	1935 mm/s
Downskin laser speed	5000 mm/s
Upskin laser speed	1100 mm/s

**Table 4:** Dimensions of samples manufactured using DMLS technology from AlSi10Mg material.

No.	a (mm)	b (mm)	No.	a (mm)	b (mm)	No.	a (mm)	b (mm)	No.	a (mm)	b (mm)
D0-T2-1	2.12	6.41	D45-T2-1	2.18	6.54	D90-T2-1	2.17	6.06	FD0-T2-1	2.33	6.02
D0-T2-2	2.20	6.38	D45-T2-2	2.17	6.62	D90-T2-2	2.15	6.08	FD0-T2-2	2.21	6.04
D0-T2-3	2.12	6.37	D45-T2-3	2.19	6.49	D90-T2-3	2.17	6.08	FD0-T2-3	2.20	6.06
D0-T2-4	2.14	6.43	D45-T2-4	2.15	6.52	D90-T2-4	2.15	6.11	FD0-T2-4	2.23	6.09
D0-T2-5	2.14	6.59	D45-T2-5	2.15	6.60	D90-T2-5	2.16	6.09	FD0-T2-5	2.22	6.01
$\bar{X}$	2.14	6.44	$\bar{X}$	2.17	6.55	$\bar{X}$	2.16	6.08	$\bar{X}$	2.24	6.04
SD	0.03	0.09	SD	0.02	0.05	SD	0.01	0.02	SD	0.05	0.03
D0-T18-1	2.03	6.32	D45-T18-1	1.97	6.53	D90-T18-1	2.01	6.12	FD0-T18-1	2.11	6.02
D0-T18-2	1.96	6.43	D45-T18-2	1.98	6.57	D90-T18-2	2.00	6.05	FD0-T18-2	2.09	6.03
D0-T18-3	1.95	6.41	D45-T18-3	1.93	6.53	D90-T18-3	1.99	6.11	FD0-T18-3	2.15	6.04
D0-T18-4	1.96	6.44	D45-T18-4	1.95	6.42	D90-T18-4	1.98	6.12	FD0-T18-4	2.09	6.07
D0-T18-5	1.94	6.39	D45-T18-5	1.97	6.52	D90-T18-5	2.00	6.15	FD0-T18-5	2.06	6.08
$\bar{X}$	1.97	6.40	$\bar{X}$	1.96	6.51	$\bar{X}$	2.00	6.11	$\bar{X}$	2.10	6.05
SD	0.04	0.05	SD	0.02	0.06	SD	0.01	0.04	SD	0.03	0.03
D0-T14-1	1.53	6.39	D45-T14-1	1.56	6.50	D90-T14-1	1.57	6.09	FD0-T14-1	1.71	6.10
D0-T14-2	1.58	6.35	D45-T14-2	1.55	6.49	D90-T14-2	1.55	6.14	FD0-T14-2	1.72	6.03
D0-T14-3	1.59	6.48	D45-T14-3	1.53	6.32	D90-T14-3	1.53	6.04	FD0-T14-3	1.71	6.05
D0-T14-4	1.58	6.48	D45-T14-4	1.51	6.39	D90-T14-4	1.56	6.10	FD0-T14-4	1.75	6.01
D0-T14-5	1.57	6.43	D45-T14-5	1.55	6.35	D90-T14-5	1.56	6.03	FD0-T14-5	1.78	6.08
$\bar{X}$	1.57	6.43	$\bar{X}$	1.54	6.41	$\bar{X}$	1.55	6.08	$\bar{X}$	1.73	6.05
SD	0.02	0.06	SD	0.02	0.08	SD	0.02	0.05	SD	0.03	0.04
D0-T1-1	1.54	6.52	D45-T1-1	1.60	6.58	D90-T1-1	1.59	6.14	FD0-T1-1	1.31	6.03
D0-T1-2	1.56	6.49	D45-T1-2	1.61	6.50	D90-T1-2	1.56	6.09	FD0-T1-2	1.25	6.05
D0-T1-3	1.61	6.48	D45-T1-3	1.54	6.43	D90-T1-3	1.57	6.11	FD0-T1-3	1.31	6.09
D0-T1-4	1.57	6.47	D45-T1-4	1.53	6.52	D90-T1-4	1.55	6.06	FD0-T1-4	1.28	6.05
D0-T1-5	1.58	6.49	D45-T1-5	1.55	6.57	D90-T1-5	1.59	6.11	FD0-T1-5	1.31	6.04
$\bar{X}$	1.57	6.49	$\bar{X}$	1.57	6.52	$\bar{X}$	1.57	6.10	$\bar{X}$	1.29	6.05
SD	0.03	0.02	SD	0.04	0.06	SD	0.02	0.03	SD	0.03	0.02
D0-T05-1	0.64	6.53	D45-T05-2	0.71	6.77	D90-T05-1	0.65	6.09			
D0-T05-2	0.64	6.54	D45-T05-3	0.67	6.75	D90-T05-2	0.65	6.08			
D0-T05-3	0.62	6.43	D45-T05-4	0.68	6.63	D90-T05-3	0.61	6.07			
D0-T05-4	0.62	6.46	D45-T05-5	0.61	6.59	D90-T05-4	0.62	6.05			
D0-T05-5	0.62	6.63	D45-T05-6	0.68	6.44	D90-T05-5	0.62	6.07			
$\bar{X}$	0.63	6.52	$\bar{X}$	0.67	6.64	$\bar{X}$	0.63	6.07			
SD	0.01	0.08	SD	0.04	0.13	SD	0.02	0.01			

**Table 5:** Values obtained from a static tensile test.

No.	Rm (MPa)	ε (mm)	No.	Rm (MPa)	ε (mm)	No.	Rm (MPa)	ε (mm)	No.	Rm (MPa)	ε (mm)
D0-T2-1	293	4.01	D45-T2-1	225	6.05	D90-T2-1	313	4.12	FD0-T2-1	212	2.55
D0-T2-2	277	3.62	D45-T2-2	234	4.74	D90-T2-2	291	3.31	FD0-T2-2	244	3.66
D0-T2-3	298	4.02	D45-T2-3	230	4.06	D90-T2-3	297	3.74	FD0-T2-3	238	3.61
D0-T2-4	280	3.49	D45-T2-4	238	4.28	D90-T2-4	304	3.78	FD0-T2-4	240	3.60
D0-T2-5	295	4.60	D45-T2-5	235	4.37	D90-T2-5	306	3.79	FD0-T2-5	248	3.59
$\bar{X}$	288.60	3.95	$\bar{X}$	232.40	4.70	$\bar{X}$	302.20	3.75	$\bar{X}$	236.40	3.40
SD	9.45	0.43	SD	5.03	0.79	SD	8.47	0.29	SD	14.17	0.48
D0-T18-1	222	3.57	D45-T18-1	232	4.23	D90-T18-1	307	4.79	FD0-T18-1	221	2.89
D0-T18-2	228	3.79	D45-T18-2	191	3.45	D90-T18-2	305	4.14	FD0-T18-2	191	2.11
D0-T18-3	225	3.29	D45-T18-3	230	4.35	D90-T18-3	302	4.44	FD0-T18-3	209	2.53
D0-T18-4	227	3.72	D45-T18-4	227	3.34	D90-T18-4	311	4.43	FD0-T18-4	238	3.97
D0-T18-5	226	3.25	D45-T18-5	237	4.52	D90-T18-5	295	3.87	FD0-T18-5	227	2.96
$\bar{X}$	225.60	3.52	$\bar{X}$	223.40	3.98	$\bar{X}$	304.00	4.33	$\bar{X}$	217.20	2.89
SD	2.30	0.25	SD	18.47	0.54	SD	6.00	0.35	SD	18.01	0.69
D0-T14-1	226	2.98	D45-T14-1	226	3.98	D90-T14-1	241	3.15	FD0-T14-1	169	2.62
D0-T14-2	217	2.76	D45-T14-2	230	4.37	D90-T14-2	242	2.92	FD0-T14-2	166	2.31
D0-T14-3	212	2.89	D45-T14-3	245	4.38	D90-T14-3	252	3.17	FD0-T14-3	169	2.33
D0-T14-4	217	3.08	D45-T14-4	239	2.75	D90-T14-4	246	3.01	FD0-T14-4	165	2.38
D0-T14-5	212	2.49	D45-T14-5	223	3.08	D90-T14-5	244	2.74	FD0-T14-5	168	3.15
$\bar{X}$	216.80	2.84	$\bar{X}$	232.60	3.71	$\bar{X}$	245.00	3.00	$\bar{X}$	167.40	2.56
SD	5.72	0.23	SD	9.18	1.12	SD	4.36	0.18	SD	1.82	0.35
D0-T1-1	213	3.40	D45-T1-1	237	4.78	D90-T1-1	235	4.73	FD0-T1-1	144	2.04
D0-T1-2	212	4.01	D45-T1-2	220	3.83	D90-T1-2	245	4.88	FD0-T1-2	157	2.13
D0-T1-3	211	4.11	D45-T1-3	235	4.56	D90-T1-3	244	4.17	FD0-T1-3	145	2.20
D0-T1-4	211	3.49	D45-T1-4	233	4.74	D90-T1-4	236	4.49	FD0-T1-4	152	2.39
D0-T1-5	212	4.13	D45-T1-5	224	4.32	D90-T1-5	240	4.96	FD0-T1-5	152	239
$\bar{X}$	211.80	3.83	$\bar{X}$	229.80	4.45	$\bar{X}$	240.00	4.65	$\bar{X}$	150.00	49.55
SD	0.84	0.35	SD	7.40	0.39	SD	4.53	0.32	SD	5.43	105.90
D0-T05-1	181	2.02	D45-T05-2	130	1.55	D90-T05-1	185	2.53			
D0-T05-2	191	2.86	D45-T05-3	151	1.69	D90-T05-2	181	2.39			
D0-T05-3	193	2.29	D45-T05-4	128	1.27	D90-T05-3	183	1.87			
D0-T05-4	194	2.34	D45-T05-5	161	1.40	D90-T05-4	199	2.65			
D0-T05-5	187	2.36	D45-T05-6	147	1.70	D90-T05-5	198	2.68			
$\bar{X}$	189.20	2.37	$\bar{X}$	143.40	1.52	$\bar{X}$	189.20	2.42			
SD	5.31	0.30	SD	14.12	0.19	SD	8.61	0.33			

**Table 6:** Surface texture parameters.

Sample Thickness (mm)	Printing Direction (°)	Sample Number	Ra	Rz	Rsk	Wa	Sa	Sz	NMP (%)
0.5	0	D0-T05	9.659	52.808	0.437	13.358	14.512	488.217	0.061
	45	D45-T05	8.895	47.999	0.662	13.174	12.482	329.670	0.051
	90	D90-T05	11.034	64.882	0.947	11.423	13.593	597.599	0.050
1	0	D0-T1	11.988	62.436	0.372	14.252	16.612	505.483	0.061
	45	D45-T1	10.562	58.557	0.217	11.395	14.397	465.435	0.034
	90	D90-T1	12.914	70.434	0.524	15.503	15.837	404.276	0.742
	parallel	FD0-T1	20.311	101.057	−0160	29.693	27.532	400.634	0.387
1.4	0	D0-T14	13.597	70.759	0.441	17.228	18.839	496.055	0.136
	45	D45-T14	11.814	65.634	0.667	14.569	15.546	469.282	0.196
	90	D90-T14	11.020	56.303	0.149	12.209	13.754	398.701	0.048
	parallel	FD0-T14	3.567	21.596	0.270	6.596	5.641	427.922	0.072
1.8	0	D0-T18	10.108	51.077	0.211	11.649	13.909	371.121	0.090
	45	D45-T18	11.102	56.607	−0.082	12.700	14.352	359.773	0.068
	90	D90-T18	11.000	55.500	0.043	10.900	19.100	213.000	0.522
	parallel	FD0-T18	4.970	30.900	0.184	8.120	10.800	267.000	0.001
2.0	0	D0-T2	10.500	58.600	−0.021	10.700	18.800	307.000	0.180
	45	D45-T2	11.701	60.632	0.415	11.378	14.975	461.854	0.092
	90	D90-T2	13.900	77.300	0.0695	13.500	22.500	429.000	0.542
	parallel	FD0-T2	3.910	22.800	0.0716	4.410	9.210	243.000	0.001

## Data Availability

Data are available upon request from the reader.

## References

[1] Etemadi E., Gholikord M., Zeeshan M., Hu H. (2023). Improved Mechanical Characteristics of New Auxetic Structures Based on Stretch-Dominated-Mechanism Deformation under Compressive and Tensile Loadings. Thin-Walled Struct..

[2] Li K., Zhang M., Hou Y., Wu Y., Ji C., He J., Jin P., Wu D., Zhu L. (2024). Multi-Scale Simulation of Residual Stress and Deformation of Large-Size Hollow Parts Fabricated by Laser-Based Powder Bed Fusion. Thin-Walled Struct..

[3] Bochnia J., Kozior T., Zyz J. (2023). The Mechanical Properties of Direct Metal Laser Sintered Thin-Walled Maraging Steel (MS1) Elements. Materials.

[4] Rudnik M. (2024). Study of Cellular Structures Built from Self-Similar Models and Repeatable Structures Manufactured by FDM/FFF Technology. Polimery.

[5] Płatek P., Rajkowski K., Cieplak K., Sarzyński M., Małachowski J., Woźniak R., Janiszewski J. (2020). Deformation Process of 3D Printed Structures Made from Flexible Material with Different Values of Relative Density. Polymers.

[6] Grzelak K., Kluczyński J., Szachogłuchowicz I., Łuszczek J., Śnieżek L., Torzewski J. (2020). Modification of Structural Properties Using Process Parameters and Surface Treatment of Monolithic and Thin-Walled Parts Obtained by Selective Laser Melting. Materials.

[7] Kozior T., Kundera C. (2021). Viscoelastic Properties of Cell Structures Manufactured Using a Photo-Curable Additive Technology—PJM. Polymers.

[8] Szczygiel P. (2024). Polymer Materials to Produce Wrist-Hand Orthoses Using the Additive Method. Polimery.

[9] Bochnia J., Kozior T., Musialek M. (2023). Flexural Properties of Thin-Walled Specimens with Square Hollow Sections 3D Printed from ABS Reinforced with Aramid Fibers. Fibers.

[10] Budzik G., Przeszlowski L., Wieczorowski M., Rzucidlo A., Gapinski B., Krolczyk G. (2018). Analysis of 3D Printing Parameters of Gears for Hybrid Manufacturing. AIP Conf. Proc..

[11] Gunnar M., Daniel M., Marcel R., Katharina S., Johannes Henrich S. Additive Manufacturing Processes In Fluid Power—Properties And Opportunities Demonstrated At A Flow-Optimized Fitting. Proceedings of the 2018 International Conference on Hydraulics and Pneumatics–HERVEX.

[12] Cooper D.E., Stanford M., Kibble K.A., Gibbons G.J. (2012). Additive Manufacturing for Product Improvement at Red Bull Technology. Mater. Des..

[13] Matthiesen G., Merget D., Pietrzyk T., Ziegler S., Schleifenbaum J.H., Schmitz K. Design and Experimental Investigation of an Additive Manufactured Compact Drive. Proceedings of the 12th International Fluid Power Conference.

[14] Anton W., Liselott E., Johan A.P., Johan Ö. (2024). Additive Manufacturing in Fluid Power with Novel Application Tohydraulic Pump Design. Int. Des. Conf.—Des..

[15] (2021). Additive Manufacturing—General Principles—Fundamentals and Vocabulary.

[16] Madej M., Piotrowska K. (2024). Characterisation of TiCN Coatings for Biomedical Applications. Coatings.

[17] Kowalczyk J., Madej M., Piotrowska K., Radoń-Kobus K. (2024). Influence of Surface Roughness on Selected Properties of the Tialn Coating. Metalurgija.

[18] (2012). Geometrical Product Specifications (GPS)—Surface Texture: Areal—Part 2: Terms. Definitions and Surface Texture Parameters.

[19] (2021). Geometrical Product Specifications (GPS)—Surface Texture: Profile—Part 2: Terms, Definitions and Surface Texture Parameters.

[20] Pawlus P., Reizer R., Wieczorowski M. (2017). Problem of Non-Measured Points in Surface Texture Measurements. Metrol. Meas. Syst..

[21] Zmarzly P., Kozior T., Gogolewski D. (2023). The Effect of Non-Measured Points on the Accuracy of the Surface Topography Assessment of Elements 3D Printed Using Selected Additive Technologies. Materials.

[22] Wang P., Song J., Nai M.L.S., Wei J. (2020). Experimental Analysis of Additively Manufactured Component and Design Guide-lines for Lightweight Structures: A Case Study Using Electron Beam Melting. Addit. Manuf..

[23] Bailey C.M., Morrow J.A., Stallbaumer-Cyr E.M., Weeks C., Derby M.M., Thompson S.M. (2022). Effects of Build Angle on Additively Manufactured Aluminum Alloy Surface Roughness and Wettability. Proc. J. Manuf. Sci. Eng.

